# Cardiovascular magnetic resonance assessment of coronary flow reserve improves risk stratification in heart failure with preserved ejection fraction

**DOI:** 10.1186/s12968-021-00807-3

**Published:** 2021-10-18

**Authors:** Shingo Kato, Kazuki Fukui, Sho Kodama, Mai Azuma, Naoki Nakayama, Tae Iwasawa, Kazuo Kimura, Kouichi Tamura, Daisuke Utsunomiya

**Affiliations:** 1 Department of Diagnostic Radiology, Yokohama City University Graduate School of Medicine, Yokohama, Japan; 2 Department of Cardiology, Kanagawa Cardiovascular and Respiratory Center, Yokohama, Japan; 3 Department of Radiology, Kanagawa Cardiovascular and Respiratory Center, Yokohama, Japan; 4 Department of Cardiology, Yokohama City University Medical Center, Yokohama, Japan; 5 Department of Medical Science and Cardiorenal Medicine, Yokohama City University, Yokohama, Japan

**Keywords:** Heart failure with preserved ejection fraction, Coronary flow reserve, Prognosis, Heart failure

## Abstract

**Background:**

Coronary microvascular dysfunction (CMD) has been proposed as a novel mechanism for the pathophysiology of heart failure (HF) with preserved ejection fraction (HFpEF). Recent studies have suggested the potential utility of coronary flow reserve (CFR) as a marker of CMD in patients with HFpEF. Phase contrast (PC) cine cardiovascular magnetic resonance (CMR) of the coronary sinus has emerged as a non-invasive method to quantify CFR. We aimed to investigate the prognostic value of CMR-derived CFR in patients with HFpEF.

**Methods:**

Data from 163 HFpEF patients (73 ± 9 years; 86 [53%] female) were retrospectively analyzed. Coronary sinus blood flow was measured in all patients, and myocardial blood flow was calculated as coronary sinus blood flow divided by left ventricular mass. CFR was calculated as the myocardial blood flow during adenosine triphosphate infusion divided by that at rest. Adverse events were defined as all-cause death and hospitalization due to HF exacerbation. Event-free survival stratified according to CFR < 2.0 was estimated with Kaplan–Meier survival methods and Log-rank test.

**Results:**

During a median follow-up of 4.1 years, 26 patients (16%) experienced adverse events. CMR-derived CFR was significantly lower in HFpEF with adverse events compared with those without (1.93 ± 0.38 vs. 2.67 ± 0.52, p < 0.001). On a Kaplan Meier curve, the rates of adverse events were significantly higher in HFpEF patients with CFR < 2.0 compared with HFpEF with CFR ≥ 2.0 (p < 0.001). The area under the curve of CFR for predicting adverse events was significantly higher than that of LGE (0.881 vs. 0.768, p = 0.037) and GLS (0.881 vs. 0.747, p = 0.036).

**Conclusions:**

CFR assessed using coronary sinus PC cine CMR may be useful as a non-invasive prognostic marker for HFpEF patients.

## Introduction

Heart failure (HF) with preserved ejection fraction (HFpEF) is as prevalent as HF with reduced ejection fraction (HFrEF) [1–4]; the prognosis of HFpEF is poor, similar to that of HFrEF [1, 5]. The prevalence of HFpEF will continue to increase as life expectancy increases [5–7]. However, effective treatment for HFpEF has not been identified because its precise pathophysiology has not been fully elucidated [8]. Coronary microvascular dysfunction (CMD) has been proposed as a novel mechanism for the pathophysiology of HFpEF [9–13]. Recent studies have suggested the potential utility of coronary flow reserve (CFR) as a marker of CMD in patients with HFpEF. The PROMIS-HFpEF (PRevalence of Microvascular dySfunction in Heart Failure with Preserved Ejection Fraction) is a prospective multicenter study that includes a large number of HFpEF patients and has shown a significant correlation between echo-derived CFR and indices of systemic endothelial function, including the reactive hyperemia index and urinary albumin-to-creatinine ratio [14]. Moreover, an autopsy study showed that coronary microvascular rarefaction is a key factor in the pathophysiology of HFpEF [12]. This evidence suggests the potential utility of CFR for evaluating disease severity in patients with HFpEF.

Phase contrast (PC) cine cardiovascular magnetic resonance (CMR) of the coronary sinus has emerged as a non-invasive means to quantify CFR [15–19]. Recent studies have shown the prognostic implication of CMR-derived CFR for coronary artery disease [20, 21] or diabetes mellitus [22, 23]. Regarding HFpEF, the CMR-derived CFR has been found to be significantly lower compared with that in hypertensive left ventricular (LV) hypertrophy and controls, and was correlated with serum brain natriuretic peptide (BNP) level [24]. Thus far, the prognostic value of CMR-derived CFR for HFpEF patients remains unknown. Therefore, this study aimed to investigate the prognostic value of CMR-derived CFR for the development of future adverse events in patients with HFpEF.

## Methods

### Study population

This retrospective, observational study included a total of HFpEF who underwent vasodilator stress CMR imaging between 2009 and 2017 in Kanagawa Cardiovascular and Respiratory Center, Yokohama, Kanagawa, Japan. The inclusion criteria included HFpEF patients who completed stress CMR tests including cine CMR, PC cine CMR of the coronary sinus, stress perfusion CMR, and late gadolinium enhancement (LGE). Indication of CMR for this study is screening of myocardial ischemia. We applied the diagnostic criteria of the European Society of Cardiology guidelines for the diagnosis of HFpEF [25]. Briefly, we defined HFpEF as follows: (1) patients with symptoms and signs of HF, (2) preserved left ventricular ejection fraction (LVEF) (LVEF > 50% on echocardiography), (3) elevated serum levels of BNP (> 35 pg/mL), and (4) objective evidence of other cardiac functional and structural alterations underlying HF (left atrial volume index (LAVI) > 34 mL/m^2^ or a LV mass index (LVMI) ≥ 115 g/m^2^ for men and ≥ 95 g/m^2^ for women, or E/e’ ≥ 13 and a mean e’ septal and lateral wall < 9 cm/s). Exclusion criteria included patients with history of prior myocardial infarction, myocarditis, hypertrophic cardiomyopathy, Anderson-Fabry disease and amyloidosis. Any evidence of persistent left-sided vena cava and low-quality CMR images were also excluded. There were 82 patients overlapping with our previous study [20]. Prognostic information was obtained using electronic medical records. Adverse events were defined as the occurrence of all-cause death and hospitalization due to HF exacerbation. Follow-up duration was defined as time of CMR scan to adverse event for HFpEF patients with events, and time of CMR scan to last follow-up for HFpEF patients without events. Clinical characteristics and echocardiography findings are information at the time of CMR scan. This study was approved by the institutional review board, and written informed consent was waived because of the retrospective design.

### CMR image acquisition

Patient scanning was performed using a 1.5-T CMR scanner equipped with 32-channel cardiac coils (Achieva, Philips Healthcare, Best, The Netherlands). The CMR protocol consisted of cine CMR, rest-stress perfusion CMR, rest-stress PC cine CMR, and LGE. Using an electrocardiogram (ECG) gated, breath-hold balanced steady-state free precession sequence, vertical long-axis, horizontal long-axis, and short-axis cine images of the LV were acquired (repetition time, 4.1 ms; echo time, 1.7 ms; flip angle, 55°; field of view, 350 × 350 mm^2^; acquisition matrix, 128 × 128; and number of phases per cardiac cycle, 20). To detect the location of the coronary sinus, axial plane cine CMR was obtained through the atrioventricular groove. The imaging plane for blood flow measurement was positioned perpendicular to the coronary sinus 1.5 cm from its ostium. During breath-holding, PC cine CMR of the coronary sinus was acquired using a vector ECG-triggered gradient echo sequence (repetition time, 7.3 ms; echo time, 4.4 ms; flip angle, 10°; field of view, 240 × 194 mm^2^; acquisition matrix, 128 × 128; number of phases per cardiac cycle, 20; velocity encoding, 50 cm/sec; and slice thickness, 6 mm). Pharmacological stress was achieved by continuous injection of adenosine triphosphate (140 μg /kg/min). First-pass myocardial perfusion CMR images were acquired with a turbo field echo sequence (4 short-axis slices/2 RR intervals; repetition time, shortest; echo time, shortest; flip angle, 40°; field of view, 360 × 324 mm^2^; acquisition matrix, 192 × 172; reconstruction matrix, 256 × 230; and slice thickness, 8 mm). After scanning of the perfusion CMR sequence  was started, gadolinium contrast (Gadopentetate dimeglumine, Magnevist, Schering, Berlin, Germany or Meglumine Gadoterate, Magnescope, Guerbet, Paris, France) was injected into the right antecubital vein at a dose of 0.05 mmol/kg and a flow rate of 4 mL/s, followed by a 20-mL saline flush. All patients were asked to refrain from caffeinated beverages for at least 24 h prior to CMR. After the acquisition of rest perfusion, gadolinium contrast was injected in a total dose of 0.15 mmol/kg. Fifteen minutes after the injection, LGE images were acquired in the same planes as the cine images using an inversion recovery-prepared gradient echo sequence (repetition time, 4.3 ms; echo time, 1.3 ms; flip angle, 15°; field of view, 380 × 380 mm^2^; acquisition matrix, 256 × 180; and slice thickness, 10 mm).

### CMR image analysis

**Fig. 1:**
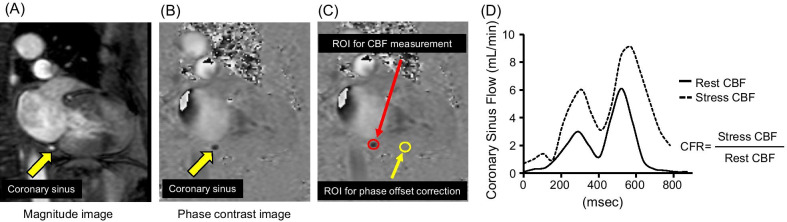
Coronary flow reserve measurement of the coronary sinus using phase contrast cine cardiovascular magnetic resonance (CMR). **A**–**C** Locations of region-of-interest (ROI) for coronary sinus blood flow measurements and phase-offset correction. **D** Representative blood flow in the coronary sinus. Coronary sinus blood flow typically peaks twice during the systolic and diastolic phases. *CBF* coronary blood flow, *CFR* coronary flow reserve

Commercially available software (Extend MR WorkSpace workstation, Philips Healthcare) was used to analyze the cine, PC, and LGE images. For the feature tracking strain analysis, dedicated software was used (Vitrea, Canon Medical Systems Corporation, Otawara, Tochigi, Japan). To assess the amount of fibrosis on LGE, enhanced myocardium was defined using the planimetry method [26, 27]. A strain analysis was performed to determine the endocardial and epicardial borders of the myocardial tissue in each cine image, and peak global radial strain (GRS) and peak global circumferential strain (GCS) were calculated using short-axis cine CMR. The peak global longitudinal strain (GLS) was calculated from the vertical long-axis and horizontal long-axis images. To calculate the right ventricular (RV) longitudinal strain, a 4-chamber view of the cine CMR images was analyzed. To quantify the blood flow in the coronary sinus, the contours of the coronary sinus were manually traced on each frame of all PC images (Fig. 1A–C). For phase-offset correction, we drew the region of interest on the myocardium separately for each cardiac phase. Coronary sinus blood flow was calculated by integrating the product of the cross-sectional area and mean velocity in the coronary sinus (Fig. 1D).

We calculated myocardial blood flow (MBF) according to the previous study [16].

- MBF (ml/min/g): Coronary sinus blood flow (mL/min) / LV mass (g).
- CFR: MBF during ATP infusion (mL/min/g) / MBF at rest (mL/min/g)

### Statistical analysis

Data were analyzed using SPSS software (version 17.0, Statistical Package for the Social Sciences, International Business Machines, Inc., Armonk, New York, USA), MedCalc for Windows (version 14.8.1, MedCalc Software, Ostend, Belgium), and R (version 3.6.3, The R Foundation for Statistical Computing, Vienna, Austria). Continuous values were presented as means ± standard deviation, and categorical values were presented as numbers (%). Normality was determined using the Shapiro–Wilk test. Normally distributed values were compared using an unpaired *t*-test, and non-normally distributed values were compared using the Mann–Whitney U test. The significance of differences in categorical variables was calculated using the Chi-squared test. The relationship between CFR and GCS, CFR and GLS, CFR and RV strain, CFR, and BNP were assessed using Pearson’s correlation coefficient. Event-free survival stratified according to CFR < 2.0 was estimated with Kaplan–Meier survival methods, and Log-rank test was used to assess the significance of difference of 2 groups. Cut-off value of CFR of 2.0 was derived according to a previous study [20]. A 2-sided p value < 0.05 was considered significant.

## Results

### Patients’ characteristics

**Fig. 2:**
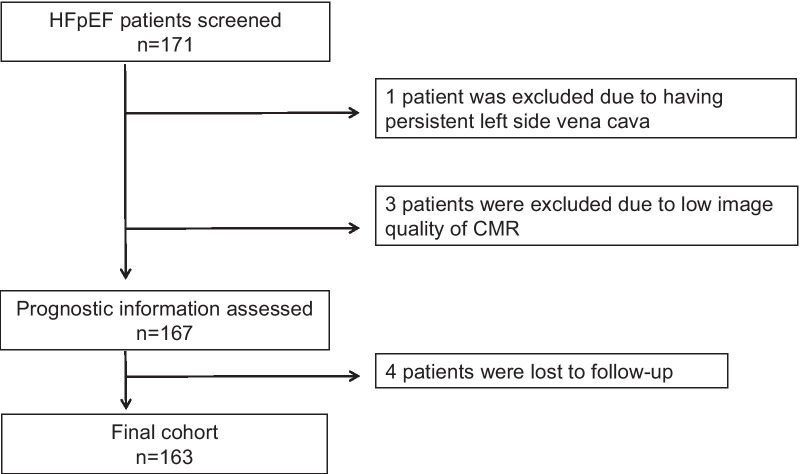
Flow chart of patient selection. *CMR* cardiovascular magnetic resonance, *HFpEF* heart failure with preserved ejection fraction

Of the 171 patients with a confirmed diagnosis of HFpEF, 163 were analyzed in this study. We excluded 1 patient with persistent left side vena cava, 3 with low image quality of CMR, and 4 without follow-up information (Fig. 2). Patient characteristics are summarized in Table 1. The mean age was 73 ± 9 years, BNP was 114 ± 80 pg/mL, and 34% of patients had a history of HF hospitalization. HFpEF patients with events had higher heart rate, high rate of history of HF hospitalization, low estimated glomerular filtration rate, and higher LAVI than those without events (all p < 0.05) (Table 1). There was no significant difference in period from diagnosis of HFpEF to CMR scan between patients without events and those with events (5.4 ± 2.1 months vs. 5.7 ± 1.7 months, p = 0.30). Fifty of 163 (31%) patients had atrial fibrillation. There was no significant difference in the prevalence of AF between patients with events and those without events (42% vs 28%, p = 0.16). CMR parameters are presented in Table 2. The mean LVEF was 64.4 ± 7.3%, and the prevalence of LGE was 45%. All hyperenhancement was located in the mid-wall or epicardial side of the LV. No patients had myocardial ischemia on perfusion CMR. HFpEF patients with events had higher %LGE, higher GCS, higher GLS, and higher RV strain compared to those without events (all p value < 0.05) (Table 2). There were significant difference in CFR (1.93 ± 0.38 vs. 2.67 ± 0.52, p < 0.001) and prevalence of CFR < 2.0 (42% vs. 3%, p < 0.001) between HFpEF with events and those without events (Table 2).

**Table 1:** Patient characteristics

	All HFpEF (n = 163)	HFpEF with events (n = 26)	HFpEF without events (n = 137)	P-value*
Age, years	73 ± 9	76 ± 8	73 ± 8	0.078
Sex, female	86 (53%)	12 (44%)	74 (54%)	0.46
NYHA class				
II/III	163 (100%)	26 (100%)	137 (100%)	–
IV	0 (0%)	0 (0%)	0 (0%)	–
Body mass index, kg/m^2^	23.5 ± 3.6	23.3 ± 3.7	23.6 ± 3.6	0.69
Heart rate, beats/min	64 ± 12	70 ± 13	62 ± 10	0.002
Systolic blood pressure, mmHg	135 ± 19	138 ± 19	134 ± 19	0.38
Diastolic blood pressure, mmHg	72 ± 11	73 ± 10	72 ± 12	0.84
History of heart failure hospitalization	56 (34%)	20 (77%)	36 (26%)	< 0.001
Smoking	4 (9%)	0 (0%)	4 (3%)	0.18
Hypertension	99 (61%)	16 (61%)	83 (61%)	0.92
Dyslipidemia	91 (56%)	14 (58%)	77 (56%)	0.82
Diabetes mellitus	41 (25%)	11 (42%)	30 (22%)	0.028
Atrial fibrillation	50 (31%)	11 (42%)	39 (28%)	0.16
Medications				
Aspirin	97 (60%)	12 (46%)	85 (62%)	0.13
Beta-blockers	62 (38%)	6 (23%)	56 (41%)	0.087
Calcium channel blockers	51 (31%)	5 (19%)	46 (34%)	0.15
ACE inhibitors/ARBs	66 (41%)	10 (38%)	56 (41%)	0.81
Statins	85 (52%)	12 (46%)	73 (53%)	0.51
Diuretics	18 (11%)	5 (19%)	13 (9%)	0.15
Blood tests				
Hemoglobin, g/dL	13.3 ± 1.3	13.2 ± 1.3	13.4 ± 1.3	0.69
eGFR, mL/min/1.73 m^2^	62 ± 13	55 ± 14	63 ± 13	0.008
BNP, pg/mL	114 ± 80	139 ± 110	109 ± 72	0.077
Echocardiography				
E/e’	14.4 ± 6.2	15.8 ± 10.7	14.2 ± 5.0	0.24
Left atrial volume index, ml/m^2^	40 ± 15	49 ± 26	38 ± 12	0.003

Data are presented as mean ± standard deviation or number (%)

*Indicates statistical significance in the differences between HFpEF patients with events and those without

*ACE* angiotensin converting enzyme, *ARB* angiotensin receptor blocker, *BNP* brain natriuretic peptide, *eGFR* estimated glomerular filtration rate, *HFpEF* heart failure with preserved ejection fraction, *NYHA* New York Heart Association

**Table 2:** Comparison of CMR parameters between HFpEF with events and those without

	All HFpEF (n = 163)	HFpEF with events (n = 26)	HFpEF without events (n = 137)	P-value*
LV ejection fraction, %	64.4 ± 7.3	62.0 ± 8.2	64.9 ± 7.0	0.29
LVEDVI, ml/m^2^	71.5 ± 17.2	72.8 ± 26.8	71.2 ± 14.7	0.66
LVESVI, ml/m^2^	25.8 ± 9.6	28.7 ± 14.7	25.3 ± 8.2	0.10
LV mass index, g/m^2^	88.0 ± 28.9	97.3 ± 27.9	86.2 ± 28.8	0.072
RV ejection fraction, %	44.8 ± 2.7	44.4 ± 2.6	44.9 ± 2.7	0.33
Presence of LGE, n (%)	74 (45%)	17 (65%)	57 (42%)	0.026
%LGE, %	5.7 ± 7.0	9.2 ± 7.7	5.0 ± 6.6	0.004
Ischemia on perfusion CMR	0 (0%)	0 (0%)	0 (0%)	–
Global radial strain, %	49.7 ± 11.8	47.0 ± 11.7	50.2 ± 11.8	0.21
Global circumferential strain, %	− 15.3 ± 3.1	− 13.0 ± 2.4	− 15.8 ± 3.0	< 0.001
Global longitudinal strain, %	− 17.4 ± 3.0	− 15.8 ± 2.4	− 17.8 ± 3.0	0.002
RV longitudinal strain, %	− 17.7 ± 3.3	− 15.5 ± 2.5	− 18.1 ± 3.3	< 0.001
Myocardial blood flow at rest, ml/min/g	1.03 ± 0.19	1.03 ± 0.19	1.06 ± 0.21	0.30
Myocardial blood flow during ATP infusion, ml/min/g	2.61 ± 0.67	2.02 ± 0.50	2.72 ± 0.64	0.15
Coronary flow reserve	2.55 ± 0.57	1.93 ± 0.38	2.67 ± 0.52	< 0.001
Coronary flow reserve < 2.0, n (%)	15 (9%)	11 (42%)	4 (3%)	< 0.001

Data are presented as mean ± standard deviation or number (%)

*Indicates statistical significance in the differences between HFpEF patients with events and those without

*ATP* adenosine triphosphate, *HFpEF* heart failure with preserved ejection fraction, *LGE* late gadolinium enhancement, *LV* left ventricular, *LVEDVI* left ventricular end-diastolic volume index, *LVESVI* left ventricular end-systolic volume index, *RV* right ventricular

### Correlation between CFR and strain parameters, BNP, %LGE

**Fig. 3:**
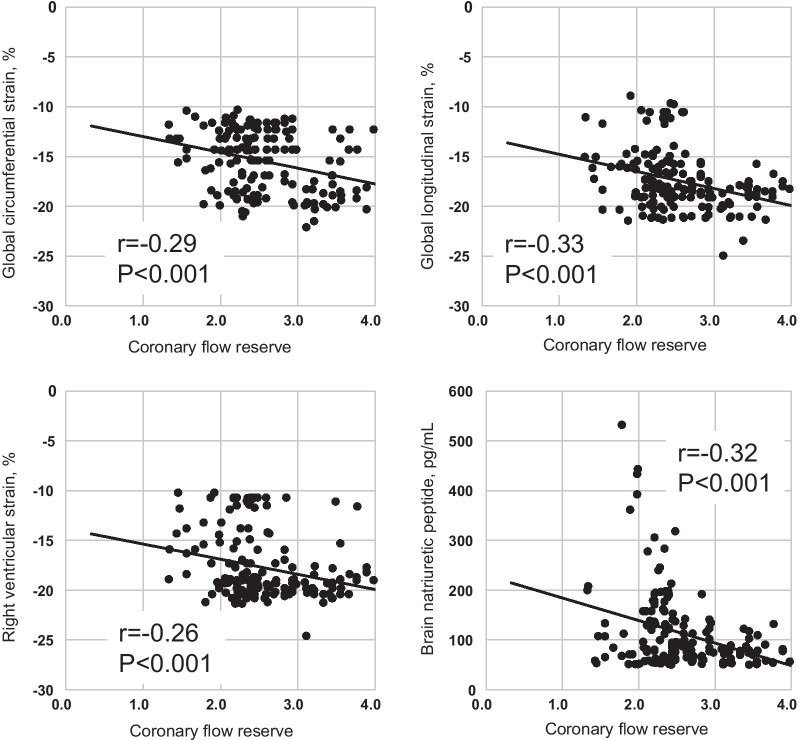
Relationship between coronary flow reserve and clinical and CMR parameters

**Fig. 4:**
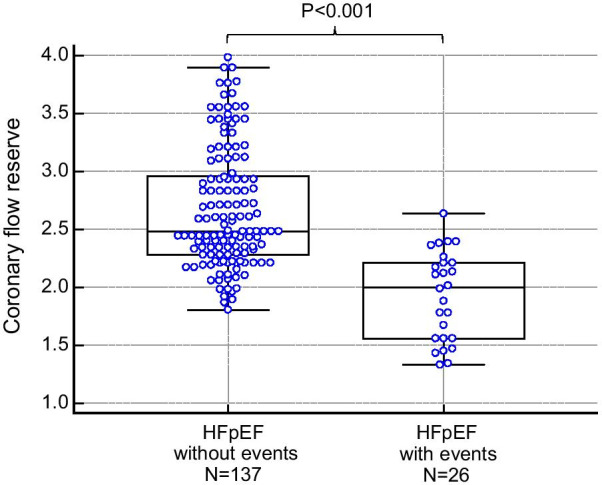
Scatter plot of CFR between HFpEF patients with adverse event and those without

Figure 3 shows the correlation between CFR and strain values and CFR and BNP. Significant negative correlations were found between CFR and GCS (r = − 0.29, p < 0.001), CFR and CLS (r = − 0.33, p < 0.001), CFR and RV longitudinal strain (r = − 0.26, p < 0.001), CFR, and serum BNP level (r = − 0.32, p < 0.001), respectively. In addition, significant negative correlation was found between %LGE and CFR (r = − 0.27, p < 0.001). Figure 4 illustrated scatter plot of CFR between HFpEF patients with adverse event and those without. CFR was significantly lower in HFpEF patients with adverse events compared with those without (1.93 ± 0.38 vs. 2.67 ± 0.52, p < 0.001).

### Prognostic value of CFR in HFpEF patients

**Fig. 5:**
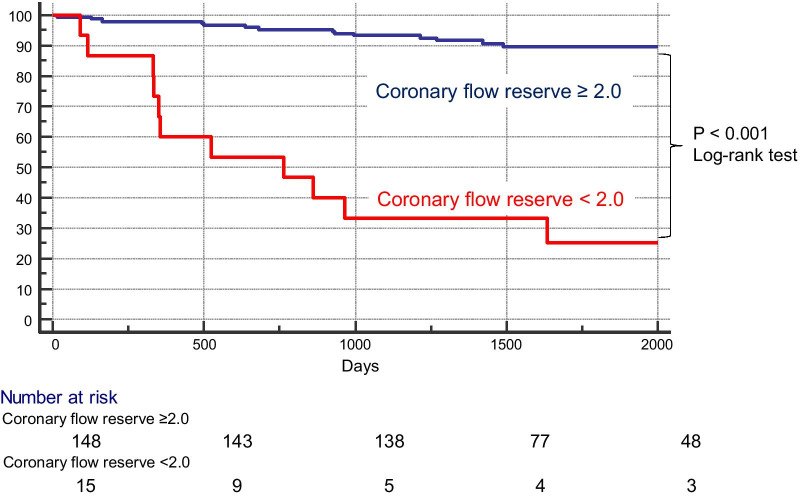
Kaplan–Meier event-free survival curves for predicting adverse events in HFpEF patients

**Fig. 6:**
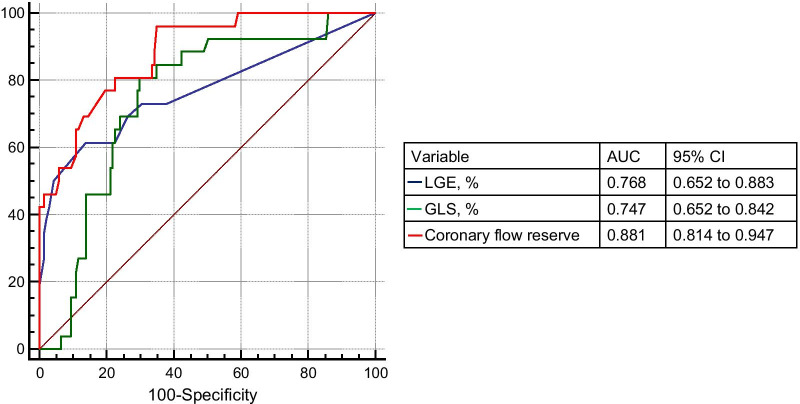
Receiver operating characteristic curves for predicting adverse events. *AUC* area under the curve, *CI* confidence interval, *GLS* global longitudinal strain, *LGE* late gadolinium enhancement

Twenty-six (16%) patients experienced adverse events over a median follow-up period of 4.1 years (cardiovascular death, n = 13; HF hospitalization, n = 13). Figure 5 illustrates Kaplan–Meier event-free survival curves for adverse events in HFpEF patients stratified by a CFR cutoff of 2.0. The rates of adverse events were significantly higher in patients with CFR < 2.0 (p < 0.001) (Fig. 5). Figure 6 shows the ROC curves of LGE%, GLS, and CFR for predicting events. The area under the ROC curve (AUC) of CFR for predicting adverse events was significantly higher than that of LGE (0.881 vs. 0.768, p = 0.037) and GLS (0.881 vs. 0.747, p = 0.036).

## Discussion

The main findings of this study are as follows. (1) CMR-derived CFR was significantly lower in HFpEF patients with adverse events compared with those without, (2) The prevalence of impaired CFR (< 2.0) was significantly higher in HFpEF with events than in those without, (3) AUC of CFR for predicting events was significantly higher than that of LGE% and GLS. These results indicate the potential utility of CMR-derived CFR for risk stratification in HFpEF patients.

In the past, several studies have suggested that CMD is an important pathophysiology of HFpEF [9–13]. In an autopsy study including 124 patients with HFpEF, HFpEF had increased mass (median, 538 g versus 335 g), more LV fibrosis (median % area fibrosis, 9.6 versus 7.1), and lower microvascular density (median 961 versus 1316 vessels/mm^2^) compared with age-matched control subjects (P < 0.0001 for all). Myocardial fibrosis increased with decreasing microvascular density in both the controls (r = − 0.28, p = 0.004) and HFpEF (r = − 0.26, p = 0.004) [12]. These results indicate that coronary microvascular rarefaction may be a key factor in the pathophysiology of HFpEF. As we excluded patients with the history of myocardial infarction, all the hyperenhancement was located in the mid-wall or epicardial side of the LV, suggesting non-ischemic etiology. Although precise mechanisms of LGE remains unclear, we believe that this LGE represents myocardial fibrosis observed in previous autopsy study of HFpEF patients [12]. PROMIS-HFpEF is a prospective multicenter study that included the largest number of 202 HFpEF patients [14]. This study has shown a high prevalence of CMD in HFpEF patients (prevalence 75%, CMD defined as CFR < 2.5 by Doppler echocardiography of the left anterior descending artery), and CFR was correlated with indices of systemic endothelial function, such as reactive hyperemia index and urinary albumin-to-creatinine ratio. These results indicated an indirect but close link between CFR and CMD in patients with HFpEF. Other small studies also showed a high prevalence of CMD in HFpEF patients (prevalence range from 37 to 76%) [24, 28–30]. In another study including suspected coronary artery disease (CAD) patients with normal LVEF who underwentpositron emission tomography) PET, impairment of PET-derived CFR is associated with diastolic function and future development of HFpEF hospitalization [31]. To date, limited data are available regarding the prognostic value of CFR for the development of adverse cardiac events in HFpEF patients. In our study, cut-off value of CFR < 2.0 showed good discrimination of HFpEF with adverse event and those without event, indicating that optimal cut-off value of CFR may be different by each modality.

PC cine CMR of the coronary sinus has emerged as a non-invasive method to quantify CFR. Theoretically, global LV blood flow can be estimated by measuring blood flow in the coronary sinus, as the coronary sinus drains approximately 96% of the total LV MBF [32]. Validation studies of this imaging technique have been performed using phantom models [33], animal experimental models using flow probes [18] and PET [16]. Recent studies have shown the prognostic importance of CMR-derived CFR for atherosclerotic diseases, such as CAD [20, 21] or diabetes mellitus [22, 23]. Additionally, this method is potentially useful for evaluating reduced CFR in patients with non-atherosclerotic diseases, including hypertrophic cardiomyopathy [15, 34], dilated cardiomyopathy [19] and HFpEF [9]. In HFpEF, CMR-derived CFR was significantly lower compared with hypertensive LV hypertrophy and controls subjects and correlated with serum BNP levels [24]. Although the precise mechanisms for alterations of CFR in HFpEF patients remain unclear, possible explanations include abnormal vascular function [35], endothelial dysfunction [36], cardiac inflammation [37], and microscopic hypertrophy and fibrosis [12]. These vascular and myocardial abnormalities observed in patients with HFpEF might be associated with impairment of CFR. In our study, significant difference of CFR was found between HFpEF with adverse event and those without, however, substantial overlap was demonstrated between 2 groups (Fig. 4). This may be explained by co-morbidities, such as hypertension, diabetes, dyslipidemia and smoking also affect the CFR value.

Recently, several CMR prognostic factors have been proposed for patients with HFpEF, such as focal fibrosis on LGE [38], diffuse fibrosis on ECV with native T1 mapping [39], RV function [40], and GLS using the feature tracking [4, 41]. Regarding GLS, a significant correlation between GLS and diffuse fibrosis quantified based on ECV with T1 mapping was found, and HFpEF with a GLS above the median of -8.5% had a higher event rate [24]. In our study, a significant correlation was found between CFR and GLS, CFR and GCS, CFR and RV longitudinal strain, CFR, and serum BNP level (Fig. 3). As most of the coronary blood flow perfuse the myocardium, correlation of CFR and LV myocardial strains could be explained by the impaired function of LV myocardial fiber due to poor perfusion. Regarding the correlation of CFR and RV strain, precise mechanism is unclear, but presumably related to pulmonary hypertension. Significant negative correlation between %LGE and CFR suggested that the link between myocardial fibrosis and coronary microvascular function in HFpEF patients. Moreover, the area under the curve of CFR was higher than that of GLS or %LGE (Fig. 5). These results indicate the clinical importance of CFR in HFpEF patients.

### Clinical implications

PC cine CMR of the coronary sinus is a non-invasive method that does not require contrast injection or radiation exposure. In this regard, this method has advantages over myocardial positron emission tomography. Even in young patients and patients with renal dysfunction, prognostic information can be obtained using this method. Additionally, because of its non-invasiveness, acquisition of PC cine CMR of the coronary sinus can be performed many times. Therefore, we can assess serial changes in the global CFR, monitoring the effectiveness of medical therapy using this method.

### Limitations

Our study has several ljmitations. First, this was a single-center, observational study with a limited number of patients. Therefore, a larger, multicenter, and more diverse study is desirable to confirm our observations. Second, the exact mechanism relating the non-invasive measurement of global CFR to increased cardiac mortality cannot be determined from this study. Third, diffuse myocardial fibrosis by T1 mapping was not performed in all subjects; therefore, the relationship between CFR and ECV was not presented in this study. Fourth, X-ray coronary angiography was not performed in all patients. However, patients with untreated CAD were excluded, and all study subjects did not have significant regional ischemia on stress perfusion CMR. Therefore, CFR would represent microvascular function rather than ischemia due to CAD in our study population. Fifth, as this study was a retrospective study, selection bias was not negligible. Although there are 82 patients overlapping with our previous paper, the target disease is totally different, HFpEF in the current study and suspected or known CAD in the previous study [20].

## Conclusions

CFR assessed using PC cine CMR of the coronary sinus may be useful as a non-invasive prognostic marker for HFpEF patients.

## Data Availability

The datasets during and/or analyzed during the current study are available from the corresponding author on reasonable request.

## Abbreviations

AUC: Area under the curve
BNP: Brain natriuretic peptide
CAD: Coronary artery disease
CBF: Coronary blood flow
CFR: Coronary flow reserve
CI: Confidence interval
CMD: Coronary microvascular dysfunction
CMR: Cardiovascular magnetic resonance
GCS: Global circumferential strain
GLS: Global longitudinal strain
GRS: Global radial strain
HF: Heart failure
HFpEF: Heart failure with preserved ejection fraction
HFrEF: Heart failure with reduced ejection fraction
LAVI: Left atrial volume index
LGE: Late gadolinium-enhancement
LV: Left ventricle/left ventricular
LVEDVI: Left ventricular end-diastolic volume
LVEF: Left ventricular ejection fraction
LVESVI: Left ventricular end-systolic volume
LVMI: Left ventricular mass index
MBF: Myocardial blood flow
PC: Phase contrast
PET: Positron emission tomography
RV: Right ventricle/right ventricular
